# Youth-centered maternity care: a binational qualitative comparison of the experiences and perspectives of Latina adolescents and healthcare providers

**DOI:** 10.1186/s12884-021-03831-4

**Published:** 2021-05-02

**Authors:** Martha J. Decker, Noelle Pineda, Abigail Gutmann-Gonzalez, Claire D. Brindis

**Affiliations:** 1 Department of Epidemiology and Biostatistics, University of California, San Francisco, 550 16th Street, 2nd floor, San Francisco, CA 94158 USA; 2 Philip R. Lee Institute for Health Policy Studies, University of California, San Francisco, 490 Illinois Street, 7th floor, San Francisco, CA 94158 USA; 3 Stanford University School of Medicine, Stanford University, 291 Campus Drive, Stanford, CA 94305 USA; 4 Adolescent and Young Adult Health National Resource Center, University of California, San Francisco, 3333 California Street, Suite 245, San Francisco, CA 94143 USA

**Keywords:** Pregnancy in adolescence, Perinatal care, Mexico, California, Patient-centered care, Qualitative

## Abstract

**Background:**

Although there is growing recognition of the importance of person-centered maternity care, the needs and perspectives of pregnant adolescents are rarely considered. The purpose of this study was to compare the maternity care experiences of Mexican-origin adolescents in Guanajuato, Mexico and Fresno, California from both youth and healthcare provider perspectives.

**Methods:**

Qualitative interviews and focus groups were conducted with a total of 89 respondents, including 74 pregnant and parenting adolescents as well as 15 providers between December 2016 and July 2017. Adolescents also completed a short demographic survey prior to participation. Transcripts in English and Spanish were coded and thematically analyzed using Dedoose software. Results were compared by location and between youth and providers.

**Results:**

Four themes emerged regarding patient-provider interactions: the need for communication and clear explanations, respectful versus judgmental providers, engaging youth in decision-making, and a focus on the age of the youth and their partners. While youth had similar perspectives and priorities in both locations, youth in Mexico reported more negative healthcare experiences than youth in California. Perspectives varied between the youth and providers, with providers in both California and Mexico identifying several structural challenges in providing quality care to adolescents. In California, challenges to supporting immigrant Latina adolescents and their families included language and translation issues as well as barriers to care due to immigration status and documentation. In both locations, providers also mentioned high patient caseloads and their own concerns about the youth’s life choices.

**Conclusion:**

Youth-centered care requires more effective and respectful patient-provider communication, where adolescents are engaged in their healthcare decision-making and delivery options. Changes in patient-provider interactions can help improve the maternity care experiences and outcomes of Latina adolescents. Healthcare systems and providers need to reconfigure their approaches to focus on the needs and priorities of adolescents.

**Supplementary Information:**

The online version contains supplementary material available at 10.1186/s12884-021-03831-4.

## Background

Adolescent pregnancy and childbirth is a key area of global health as it represents a critical intersection between issues of maternal and child health, gender equity, and social determinants of health such as educational attainment [1]. The adolescent pregnancy rate in Mexico remains one of the highest in the Americas at 71 births per 1000 women ages 15–19 [2]. In the United States, Latina adolescents have among the highest rates of adolescent pregnancies of any racial or ethnic group [3]. In California, nearly 75% of all adolescent pregnancies are among Latina youth [4] with approximately one quarter of adolescent births to foreign-born Latinas [5].

Professional policy statements for pediatricians and gynecologists highlight the unique needs of pregnant adolescents and emphasize the importance of tailoring perinatal care to the developmental needs of this population [6]. While there is a growing body of research regarding youth-friendly health services and person-centered care, virtually none of these focus on pregnant adolescents or their perspectives [7, 8]. Further, little is known about how adolescents perceive their maternity care experiences, how their perspectives compare to those of their healthcare providers, or if this varies by sociocultural context.

In addition, some populations may face additional barriers to accessing person-centered and quality care. Previous research has found that certain groups including adolescents, unmarried women, women of low socioeconomic status, and women belonging to ethnic minority groups are at a higher risk of experiencing disrespect and abuse in the provision of care [9]. In Mexico, young pregnant women with lower socioeconomic and educational levels and those living in more rural areas are less likely to receive the standard of care [10]. Similarly, Latina adolescents in the United States are more likely to have inadequate prenatal care compared to white adolescents or older Latinas [11].

This article explores the experiences of Mexican-origin adolescents receiving maternity care in two different healthcare and cultural contexts – one in which they are in their home culture of Mexico and one in which they are an immigrant group in California. It also compares the perceptions of the adolescents to healthcare providers in both contexts. Through this comparison, the findings can be further delineated to identify those which may be more universal to adolescents’ experiences and perceptives of maternity care and which are more context-specific.

The Institute of Medicine defines patient-centered care as, “providing care that is respectful of, and responsive to, individual patient preferences, needs and values, and ensuring that patient values guide all clinical decisions” [12]. Person-centered maternity care (PCMC) elaborates on this definition by focusing on reproductive and maternal care and recognizing that respect and dignity, provider responsiveness, communication, and patient interpersonal treatment are all integral pieces of the patient experience [13, 14]. Prior research from around the world has found positive associations between PCMC and maternal and newborn health outcomes [15, 16]. PCMC has also been associated with higher levels of patient satisfaction, and patient health outcomes [17].

While there is a growing emphasis on the importance of PCMC for maternal and infant health, adolescents’ needs and perspectives are rarely considered. Past studies have found that young mothers report more pain, fear, and lack of control during their delivery experiences than women of other age groups and that many adolescent patients describe their delivery experience as traumatic [18, 19]. Research on patient-provider interactions among adolescents in general primary care clinics has highlighted youth’s concerns regarding confidentiality and judgmental provider attitudes [20, 21]. One systematic review that explored youth’s general healthcare experiences, found eight domains linked to youth’s positive experiences of care including accessibility of services, staff attitudes, communication, and youth involvement in healthcare [8].

Little is known about the maternity care experiences of pregnant and parenting Latina adolescents in either the United States or Mexico. Recent research documents that Mexican immigrants experience a reduction in healthcare access after they migrate to the United States and continue to face more challenges in accessing healthcare health care as compared to the non-migrant population [22]. A small study of Latina youth in the United States found that over 95% of the sample experienced personal and institutional barriers when attempting to access prenatal care [23].

Given that pregnant and parenting adolescents are at-risk for receiving sub-optimal health services and that Latinas affected by migration are more likely to have limited prenatal care, a greater understanding of the healthcare experiences of pregnant and parenting Latina adolescents is needed. Notably absent are studies that provide the perspective of both youth and their healthcare providers, as the providers’ perceptions and context may impact how they interact with pregnant youth and their delivery of services. Thus, the purpose of this study is to compare the perinatal experiences and perspectives of Mexican-origin adolescents and their healthcare providers in two distinct sociocultural and healthcare contexts — Mexico and California.

## Methods

### Setting and study population

Respondents were selected from several communities in Fresno County, California, and Guanajuato, Mexico. Guanajuato, Mexico, was selected as a traditional point of origin for migrants to California and Fresno, California, was selected as a primary point of arrival for Mexican immigrants. Both settings are major agricultural regions and reflect a mixture of urban and rural communities.

Female youth were eligible if they were either pregnant or post-partum (within 12 months of delivery), spoke English or Spanish, and were 14–20 years of age. Youth respondents in California were required to either have migrated from Mexico or have at least one parent who had. A convenience sample of youth participants in Mexico were recruited from public hospitals and clinics, while youth in California were recruited from several community-based organizations serving pregnant and parenting youth. Study researchers had established relationships with clinics and organizations in both communities through other projects, and staff at these sites recruited youth to participate when they sought services at these sites. Health providers in both locations were selected who had experience providing care to pregnant and/or parenting Latina youth. Providers were purposively selected from multiple institutions in each location to gather a range of professional backgrounds and experiences, including nurses, social workers, health educators, and physicians. Recruitment and data collection stopped when saturation of responses was complete [24].

### Study instruments

Qualitative data was collected using focus groups and in-depth interviews to gather individual histories and experiences as well as build on group-generated  ideas [24]. All tools were developed by the researchers for this study and are not under license. Interview and focus group tools, shown in Additional files 3, 4 and 5, were pilot tested and modified accordingly.

#### Focus groups

Focus groups were conducted with an average group size of five young women who were either pregnant or up to 12 months post-partum. Key topics covered in the focus groups included participants’ experiences during perinatal care and services available for pregnant/parenting youth. Focus groups averaged an hour in length.

#### In-depth interviews with female youth

To discuss potentially sensitive topics in a more confidential setting, select youth completed an in-depth interview focusing on their personal experiences receiving perinatal care, any complications related to pregnancy and delivery, and the migration histories of their families. Interviews averaged 22 minutes in length.

#### In-depth interviews with health providers

Interviews with health providers focused on service protocols and guidelines that they follow with pregnant and parenting youth, as well as their opinions about pregnancy and care among that specific population. Each interview was approximately an hour long.

#### Demographic survey

Adolescent participants completed a short survey, shown in Additional file 2, containing questions about their socio-demographic characteristics and reproductive and pregnancy histories prior to the focus groups and interviews.

The study protocol was approved by the the Institutional Review Board for the Human Research Protection Program of the University of California, San Francisco (approval number 16–20062), and the Committee of Investigation of the Secretariat of Health in Guanajuato, Mexico.

### Data collection

A binational team of trained and experienced researchers from Mexico and the United States conducted all focus groups and interviews in the language preference (Spanish or English) of participants between December 2016–July 2017. Researchers, four females and one male, represented two different institutions and a range of disciplines including social epidemiology, public health, medicine, and ethnography. Researchers were interested in conducting this study to identify ways to improve care for adolescents. Prior to each interview and focus group, researchers introduced themselves, discussed the purpose and process of the study, and provided written consent forms with details about the study. Active parental consent was not required for this study per state and institutional guidelines. Before the focus groups and interviews, youth participants completed a short demographic survey.

Focus groups and interviews were typically held in classrooms or clinic meeting rooms. Focus groups and interviews in Mexico took place in public hospitals and clinics while those in California were primarily conducted in community-based organizations. Only researchers and participants were present during focus groups and interviews. In recognition of their time and input, respondents received a $20 gift certificate in California while in Mexico, participants received infant supplies, such as diapers, per local institutional recommendations.

A note-taker typed responses and the focus groups and interviews were audio-recorded. Focus groups were professionally transcribed verbatim in Spanish or English, and research staff transcribed interviews.

### Data analysis

This study used a modified form of grounded theory in which a set of potential concepts and relations were identified based on the research’s initial socio-ecological model [25] and elements of patient-centered care as well as through themes inductively identified from the data [26]. This approach maintains the essential elements of grounded theory including an iterative and reciprocal data-theory relationship. Researchers developed a mixed coding system that used a combination of structural and emerging coding [27]. An initial list of codes using the main research questions was created with additional codes added based on further review of the transcripts and fieldwork notes [28]. The final codebook contained 53 codes.

Three researchers coded the focus groups and interviews in the original language, with a fourth researcher reviewing for inter-coder consistency. Throughout the coding process, the researchers met weekly to review the process, clarify codes, and make minor modifications to the codebook to improve reliability. In addition, a random sub-sample of nine interviews and three focus groups were coded by two separate researchers (blinded to the coding of each other) and inter-coder reliability tests were performed to ensure the consistency of the coding, obtaining an average Cohen’s kappa value of 0.88. If a quotation was coded differently, researchers discussed the options and jointly agreed to a coding allocation. The qualitative coding was conducted using Dedoose [29] and the survey data was analyzed in Excel.

Four researchers identified patterns and relationships in each code and across codes and extracted relevant themes. Additional sub-codes were developed based on the themes that emerged during analysis. Commonalities and differences in themes between locations (California and Mexico), and between youth and providers, were subsequently assessed. Stakeholders received a summary of findings and were invited to provide feedback.

This study follows the consolidated criteria for reporting qualitative research, shown in Additional file 1 [30]. Any Spanish quotations used in this manuscript were translated by one bilingual researcher and verified by a second bilingual researcher.

## Results

A total of 74 youth and 15 providers participated in the study, with greater numbers of Mexican youth (*n* = 49) participating compared to the California sample (*n* = 25). A slightly larger number of providers were interviewed in California (*n* = 9) compared to Mexico (*n* = 6) (Table 1). Most youth participants in Guanajuato, Mexico, received prenatal care from governmental neighborhood clinics and delivered in the district maternity hospital. In contrast, the participants in Fresno, California, received prenatal care from a variety of clinics and delivered in various local hospitals.

**Table 1:** Focus group and interview participants, by location

	California	Mexico	Total
Number of focus groups	5	6	11
Focus groups total participants	20	39	59
In-depth interviews with youth	5	10	15
In-depth interviews with health providers	9	6	15
**Total youth and provider participants**	**34**	**55**	**89**

Providers in California included nurse practitioners, medical assistants, health educators, and youth health program directors. Mexican providers included physicians, nurses, social workers, and health educators. Although one male provider was interviewed in California and one in Mexico, the majority were female. Additionally, slightly over half of the providers in California were of Latino descent.

According to the information provided in a brief survey prior to participation, youth participants in both settings were of similar age (17 years) and all were either pregnant or new parents. However, the populations differed in other aspects (Table 2). In California, 88% of youth participants were parenting, while in Mexico, 55% of participants were parenting at the time of their focus group or interview. Of those who were parenting, three participants in Mexico had two or more children compared to one in California. Among parenting youth, nearly 50% in Mexico reported delivering via cesarean section (C-section) compared to 14% in California.

**Table 2:** Demographics of youth focus group and interview participants by site

	**California (*n*** **= 25)**	**Mexico (*n*** **= 49)**
**Age (average)**	17.5 years	17.2 years
**Relationship status**		
Single	9 (36%)	3 (6%)
In a relationship, but not living together	6 (24%)	8 (16%)
Married/living with partner	10 (38%)	38 (78%)
**Current pregnancy status**		
Pregnant	3 (12%)	22 (45%)
Parenting	22 (88%)	27 (55%)
**Ever previously pregnant**		
Yes	4 (16%)	7 (14%)
No	21 (84%)	41 (84%)
**Delivery method of parenting participants**	**California (*n*** **= 22)**	**Mexico (*n*** **= 27)**
Vaginal delivery	13 (59%)	9 (33%)
C-section	3 (14%)	13 (48%)
Missing information	6 (27%)	5 (19%)

### Youth and provider perspectives on maternity care

Four cross-cutting themes emerged from youth and providers in both locations regarding patient-provider interactions: communication and clear explanations, respectful or judgmental providers, engagement in decision-making, and a focus on the age of the patient and their partners. Perspectives and concerns regarding maternity care experiences varied widely between the youth and providers, regardless of location. While youth generally focused on the positive and negative characteristics of their providers, the providers were more likely to focus on structural challenges such as limited appointment times, institutional support, and insurance and other documentation issues. Although similar preferences and concerns emerged from youth in both locations, negative experiences were more common and often more severe for youth in Mexico.

### Patient-provider communication and the need for clear explanations

#### Youth perspectives

Youth in both locations described the importance of clear communication and explanations from providers, although more youth in Mexico emphasized wanting explanations of clinical assessments than in California. Many youth in both California and Mexico described interactions with providers where they were not given clear explanations of what was happening and what clinical decisions were being made. Youth also described frustration with asking their health providers questions and not receiving sufficient answers. One participant said:

*“I had asked them what's the infection that [my baby] has; I wanted them to explain it better to me …. They made me feel really frustrated that they weren't really explaining it to me ... I tried asking the nurse that was in the NICU and they didn't know how to explain it to me … I do remember asking them and asking them.” (Youth focus group, California)*

In California, a few youth mentioned feeling fearful of asking questions of their providers because the provider made them uncomfortable. Many youth in Mexico indicated fear around the delivery of their babies, in particular because they did not know what to expect since providers and clinic staff did not always explain things to them.

*P1: “The truth is, that I was scared as well. I went there without having pains, without anything, but when they put me with the other patients, and I saw how they were feeling, I started to feel nervous.*

*P2: I was scared, too. I was thinking about what they were going to do to me.” (Youth focus group, Mexico)*

Many youth, but only in Mexico, recounted negative experiences of providers deliberately ignoring them and asking to speak with their parents instead of directly interacting with the youth. One participant explained how she learned that she would need a C-section:

*“They explained it to my mom first and then my mom came to tell me that they had to do a C-section [and] to not be scared. But it was my mom who explained it to me.” (Youth interview, Mexico)*

One participant with a positive experience said:

*“They explained everything to me. When I had the baby, they also explained it all again to me.” (Youth interview, Mexico)*

#### Provider perspectives

Providers in both locations identified several challenges in communicating effectively with their adolescent patients, including personal opinions and structural issues. A few providers in both locations expressed a desire to stress the risks involved in adolescent pregnancy, with one provider stating:

*“I give them an overview of the possible complications of an adolescent pregnancy, so that they can take into account what can happen and see that the decision they made is serious. Sometimes I tell them, it may sound unpleasant, but I tell them—look, there is preeclampsia, diabetes, premature birth and because of your age, it is very common that it can happen … We agree that although this is not the age to get pregnant, that we will do everything possible to detect any problem early and that they know the signs and or symptoms that might emerge.” (Provider, Mexico)*

One medical assistant in California discussed how they facilitate communication between doctors and the young women as many adolescents feel uncomfortable speaking directly with their provider:

*“Sometimes they don’t open up to the doctor, but they will open up to us. Sometimes we’ll have to let the doctor know if there’s, you know, something that comes up or there’s something that they need to address.” (Provider, California)*

In California, many providers described language barriers when communicating with immigrant youth and their families, particularly when needing to convey difficult information. Providers also discussed the need for language services other than Spanish since many recent migrants from Mexico only speak indigenous languages. As one provider said:

*“There’s even some [patients] that speak only Mixteco [indigenous language of Mexico] … those are the ones that need the most care, the most help … It’s a very guarded community. So, what stays within their community, happens between their community and hush, hush, don’t say anything. So, it’s very hard if they bring an interpreter to get things out of them, because they’re not going to tell us. If not, you know, it’s kind of hard to be translated when the parent or a brother or a sister is present because they’re very guarded on what they say.” (Provider, California)*

Several providers in California also mentioned that concerns about insurance and immigration status limited interactions with patients.

*“I think especially the ones that have come here undocumented, or illegally, you know the fear of accessing care …. A lot of them will fly very low, under the radar and don’t take services. You know they might tap into MediCal for the baby once the baby is born but will take very minimal services out of fear of having to disclose their immigration status.” (Provider, California)*

### Respectful or judgmental providers

#### Youth perspectives

Youth most frequently mentioned the personal characteristics of their provider when reflecting on their maternity care experiences. Several youth in both locations appreciated feeling supported by their providers and receiving “normal” treatment. As one youth in Mexico stated:

*“They treated me well … since the start of my pregnancy until I recovered. Yes, they treated me well, really. And they were very careful to think about everything.” (Youth interview, Mexico)*

A few youth in California described feeling comfortable when providers and staff were non-judgmental, respectful, and kind. Conversely, only one youth in Mexico mentioned feeling comfortable with their provider. One participant said:

*“They're really nice. They talk to you, they mostly focus on you and the baby … I felt very supported and not judged because I was ... a teen mom.” (Youth focus group, California)*

One youth in California appreciated when providers treated them in an approachable manner and used their names:

*“I would walk in and they would already know my name so that’s why I like going there. Some doctors, they just see you as a patient and others, they would actually know my name when I walk in.” (Youth interview, California)*

A few youth in both California and Mexico described providers who were rude and judgmental in their interactions with them. One participant said:

“*The doctor didn’t want to look after me, and even threw my papers at me; he was very rude. He threw the papers at me and demanded that my mother be called because I was overreacting.” (Youth focus group, Mexico)*

Often, youth in Mexico felt they could not express pain or discomfort because providers would get angry and pay less attention to them. Concerns about or experiences of pain, particularly around delivery, were the most common negative issue among youth in Mexico. In contrast, only a few youth in California mentioned pain. As one youth stated:

*“If you are dramatic and loud, they hardly pay attention to you. There was a girl who had a one-year-old and she was pregnant again, but she was screaming [during delivery] as if someone were killing her, so they didn’t really pay attention to her, because she was overreacting.” (Youth focus group, Mexico)*

Many youth in Mexico, but only one participant in California, acknowledged the variation of experiences within the same hospital or clinic depending on the provider. One participant described the different providers at one hospital:

*“The truth is there are doctors that are mean, but the doctor who delivered my baby was very good to me. It depends on which shift you are on … because the night shift, truthfully, they are all very careless. I did not like the night shift because they made fun of the girls.” (Youth focus group, Mexico)*

While some youth in California discussed having negative experiences with their initial providers, all of them felt like they could switch providers. Indeed, many youth in California described switching to a provider they liked better. One youth described her experience:

*“I switched [doctors]. My [new] doctor was really nice. He told me everything. He explained everything to me, what I could give [my baby to eat], after I had my baby and stuff like that. I really liked my doctor, he was nice. It was just that one doctor [who was bad].” (Youth focus group, California)*

Many youth in California also mentioned preferring female providers and would change doctors if they were assigned a male provider.

#### Provider perspectives

Although mentioned infrequently, a few providers were aware of the criticisms from patients around lack of respectful, patient-centered care. Providers most often attributed their inability to provide patient-centered care to structural constraints, though some also recognized their own negative opinions regarding adolescent pregnancy and their patients’ life choices.

Providers in both countries expressed concern about patient caseload and the resulting limits on how much time they could spend per patient. In Mexico, one provider discussed how their caseload and the insufficient number of doctors limited the time they could spend with individual patients:

*“We have to provide care to everyone, and if we cannot provide specialized care to a patient, then we just get stuck. The truth is that we do not have much time to be focused on an actual medical appointment, and evaluate the entire situation around a particular patient.” (Provider, Mexico)*

Providers in both locations also acknowledged their own underlying concerns about adolescent pregnancy and its consequences. Many providers in California and a couple of providers in Mexico focused on the tension they felt about what they perceived as being for the patient’s “own good.” Providers discussed how personally difficult it is for them when they strongly disagree with the life decisions made by their pregnant and parenting adolescent patients. For example, one clinical provider in California discussed the issue of staying in school:

*“So I think the difficult thing for me as a provider and the clash that I have sometimes is that with teens, I want them to remain in school, and I sometimes get pushback, you know, because here I am, this educated white person coming in telling her how to raise her child. You know this is not what goes well, so you have to be really careful with that.” (Provider, California)*

Similarly, one health educator in Mexico said:

*“Sometimes I tell people, ‘We are sorry, it’s just that we sometimes worry.’ … We can’t avoid it because they worry us. That is, sometimes it does cause anger, how you put your life at risk, when we are offering you, we are offering everything to plan your family and you don’t take advantage of it … [Then] I remember that it isn’t my responsibility, that my responsibility is to just offer [options] and information, that I can’t force them to make decisions, even for their own good.” (Provider, Mexico)*

### Shared decision-making

#### Youth perspectives

In general, youth in California expressed more agency around decision-making, especially around their delivery and clinical care, compared to youth in Mexico. When asked whether their provider had discussed delivery options with them, one youth in California explained:

*“I talked about it and I had made up my mind ... I had barely got transferred to [the hospital] when they asked ... so they could write it down and keep the information. So on delivery day, I had said I wanted normal [delivery] and when I went in, I was already asking for a C-section. I'm like ‘I can't!’ And they're all like, ‘oh you can. You're ready to open ...’ … They ask you about it.” (Youth focus group, California)*

In contrast, youth in Mexico were rarely asked their opinion about their clinical care and most felt they could not disagree or question clinical decisions. For example, many youth described having never discussed the different delivery methods or their care plan with their providers. As one youth described:

*“I hadn’t thought about … I’d never considered … well, how I wanted it to happen, normal [delivery] or C-section.” (Youth interview, Mexico)*

A common experience for youth in Mexico was arriving at the maternity hospital and being turned away by providers who thought it was too early in the labor process. Many youth in Mexico described feeling frustrated about being sent home after going into labor, stating that providers ignored their requests and refused to admit them into the hospital. One youth ultimately gave birth outside the hospital because they would not admit her:

*“I started to feel bad on a Friday. I went to the Maternity Hospital on that Saturday and they told me I was not ready yet, I was 1 cm dilated. On that Sunday, I could not take the pain any longer, and they told me that I still had one week left. The doctor told me to stop bothering him … that I still had one week left … I was assisted by a midwife when I was delivering, because in the Maternity Hospital, they did not want to take care of me.” (Youth focus group, Mexico)*

#### Provider perspectives

While some youth expressed frustration about being ignored or having their concerns dismissed, particularly in Mexico, only a few providers mentioned the lack of youth engagement in decisions related to their care. Some providers in California, but none in Mexico, discussed the power imbalance that exists between providers and adolescent patients. They noted that many youth do not feel empowered to make their choices known, especially around the time of delivery, or that the youth’s opinions are disregarded by medical staff. As one health educator recounted:

*“I think what we hear from their experience during delivery especially, that it’s sort of like happening outside of them, there’s not a lot of, ‘well what do you want and what do you need?’ … And some are like really into this birth plan and ‘I’m going to do this!’ And they would go and the nurses would be like, ‘Uhhh!’ Just not even respectful of the fact that they had made this birth plan and they wanted these certain things … It’s just ‘you don’t know,’ ‘we’re doing this for you’. I hear a lot of, ‘nobody explained to me anything. They just did their things their way’. And they just feel like they weren’t in the loop.” (Provider, California)*

These interactions may make youth feel more disempowered within the healthcare system. Many youth don’t know that they can question or have an opinion on the care that they receive. As two health educators explained:

*P1: “Most of them do not [feel empowered], they are going to do whatever you tell them to.*

*P2: Yeah … they just said that’s what the doctor told them to do.” (Providers, California)*

Another challenge related to decision-making is that medical staff may have preconceived ideas about youth, which can affect the services they deliver. One provider in California explained:

*“Their [youth] experience at the birthing center with nurses, they get this feeling of impatience with the nurses because [the nurses] assume they know nothing. It’s been a big complaint, or they have been upset that [the nurses] immediately give the baby a bottle of formula and didn’t give her the opportunity to nurse the baby first.” (Provider, California)*

### Focus on age of adolescent and partner

#### Youth perspectives

Many youth in California and a couple of youth in Mexico stated that clinic staff and providers focused too much on their age or the age of their partners. During one focus group, two participants shared:

*P 1: “My doctor told me I was too young and I was like, ‘okay.’”*

*P 2: “Mine too. He didn't tell me, he just looked at me and was like, ‘you're 15!?’” (Youth focus group, California)*

Similarly, one participant in Mexico said:

*“Well, the first time [the doctors] saw me they said, ‘How come you will be turning 14 and will already have your first child?’ At the beginning, they told me that I was going to be playing with baby dolls, but that now I had to feed them for real.” (Youth interview, Mexico)*

In California, some youth mentioned appreciating when providers did not make comments regarding their age and instead treated them like “normal” patients. One participant explained that clinic staff:

*“ … didn’t ask me anything [about my age]. They just checked me and … she was asking me normal questions.” (Youth focus group, California)*

Many youth in California, but none in Mexico, described uncomfortable situations when providers asked questions regarding the father of the child. One participant said:

*“When my doctor seen my age, he kept asking me a lot of questions and he was rude and he kept asking for who was the dad and I was like, ‘Well, I don't know,’ because I wasn't sure what to say. ‘Well how old is he?’ I was like, ‘I don't know’. I didn't want to reply. He was like, ‘Is he 13, 60, 30? Do you not know how old?’ I don't know. So he made me feel so uncomfortable.” (Youth focus group, California)*

One youth in California discussed being wary of providers’ questions because of the potential impact requiring reporting of their partner’s age:

*“They ask a lot of questions ... a lot. My sister-in-law when she was pregnant, the same clinic called the cops on her. So, my brother had to go to court and everything … She was 13 and my brother was 15 … and he ended up still getting locked up for that.” (Youth focus group, California)*

#### Provider perspectives

Similar to the youth respondents’ concerns, providers in California focused on the negative influence of mandated reporting. This legal requirement to report sex by an adult with a minor restrained patient-provider communication. Health providers acknowledged that pregnant and parenting adolescents are unlikely to be fully forthcoming given their status as mandated reporters. One provider described feeling conflicted about their role as a mandated reporter and that the decision to report is not always as clear-cut as it might seem:

*“We didn’t ask about those things, even though we’re mandated reporters … like, I have a 23-year old dad here, who really loves this 16-year old girl, and they’re a family and he is a good guy and he’s providing for them and that would separate that family … There were some times that it was very morally conflicting because, on one hand, you know and so you’re trying to constantly get information, but not too much information. But, then also you know you do identify those times where you’re like this is a total predatory relationship. And then you do make reports.” (Provider, California)*

Similarly, another provider discussed a particular patient who was reported to Child Protective Services (CPS):

*“She was about second trimester when she came in … she was Mixteco [indigenous people of Mexico] and she was married, but she’s still a minor so CPS got involved and took her and gave her a foster mom. That was the hardest because she got taken from her husband [and] from everything that she knew.” (Provider, California)*

In Mexico, two providers mentioned the age of the partner, but in both cases, they stated the partner was around the same age as the young mother. Although mandated reporting laws exist in Mexico, none of the providers mentioned them. A couple of providers in California mentioned that some young women are in unhealthy relationships with older men and can feel pressured to stop using contraceptives and have more children. As one provider stated:

*That’s probably one of our obstacles, especially since the boyfriends are older... They are like ‘well he’s ready to have another baby’ and it’s a struggle because they forget about what they want … I think it’s a lot about control. You know like ‘the more babies I have with you, the less likely you are going to be to leave’.”* *(Provider, California)*

## Discussion

This qualitative study is the first to our knowledge that compares the maternity care experiences of adolescents in the contexts of two countries and from both youth and provider perspectives. Although the healthcare and sociocultural contexts differed, youth in both locations had similar perspectives and preferences regarding their care and provider interactions. In contrast, different issues emerged from the vantage point of providers, with a focus on several structural barriers, such as limited time per patient due to high caseloads. In California, language barriers, mandated reporting requirements, and general barriers to accessing healthcare, particularly among recent immigrants and those from indigenous groups, created additional difficulties. Improving working conditions and ensuring an adequate healthcare infrastructure may improve the care patients receive [31].

Our findings on the importance of youth-centered maternity care fill a gap between existing research on person-centered maternity care and youth-friendly health services. As with prior studies, this research highlights the need for effective patient-provider communication and shared decision-making among adolescent patient populations [32, 33]. Youth described frustration when receiving unclear explanations of what was happening in their clinical care, while providers described tensions around communicating what was for the patient’s “own good”. As Teagle and Brindis previously found, “inadequate patient-provider communication may represent one of the single most important nonfinancial barriers to care” for adolescents [34].

Power imbalances between the adolescent patient and clinicians and a lack of agency in decision-making were also found in both locations. Particularly in Mexico, youth often had little choice over their provider or control over their delivery and worried about expressing pain in front of providers. This mirrors similar findings in Mexico that identified authoritarian behaviors by providers and passivity among patients, especially when patients were perceived to belong to a lower social class [35]. Adolescents may be more likely to experience discrimination and disrespect by providers due to the intersectionality of their young age, poverty, and immigration status [36, 37].

Our findings suggest that the maternity care experiences of Latina adolescents can be improved through a commitment to person-centered practices and more effective patient-provider communication, where the adolescent patient is treated respectfully, included in their healthcare decision-making, and informed of their rights and options. Medical and nursing schools should incorporate curricula and assessments focused on equitable care as well as strive to diversify their student bodies and faculty. Healthcare professionals working with youth should receive training in motivational interviewing and develop skills to address their unconscious biases to reduce lecturing and increase patient self-efficacy. A recent review of practices to promote respectful care found that skills-based training on values, transforming provider attitudes, and interpersonal communication showed promise [31]. In addition, hospitals and clinics should develop robust accountability systems to ensure that the experiences of youth are assessed and valued.

Similar to research in other settings and to literature in both youth-friendly services and person-centered care, youth in both countries preferred respectful, non-judgmental care [38, 39]. Youth often felt judged by providers who focused on the patient’s young age or were made to feel uncomfortable by providers who questioned them about the father of the child. Recent research shows some providers may disrespect their patients as a coping mechanism in high stress, limited-resource healthcare environments and disregard the patients’ preferences when they do not conform to the physician’s clinical experiences or assumptions [37, 40]. In California, concerns about mandated reporting requirements of the partner’s age appeared to act as a barrier to establishing inter-personal trust. Currently, California health care providers are not required by law to ask their minor patients the age of their sexual partners, but are expected to ask relevant questions based on each provider’s professional judgment and to report situations with significant age differences [41]. Conflicting interpretations of similar legal requirements in Mexico may also cause confusion for clinicians [42]. Our findings suggest that health providers need further clarification and training regarding the legal requirements of mandated reporters and how to appropriately inquire about the partners of adolescents.

While these results identify several common themes in both locations, it also highlights some context-specific considerations. The desire for respectful care appears universal; however, adolescents in Mexico generally reported less communication or consultation with their providers during delivery and more instances of disrespect than Latinas in California. Unfortunately, disrespectful maternity care remains common throughout the world, particularly for younger women and those with lower income [35, 43].

This study had several limitations. The generalizability of this study’s findings is limited by the specific healthcare contexts of the two locations and the type and number of providers interviewed. In Mexico, most adult respondents were healthcare providers working in a hospital setting, while in Fresno, respondents represented a broader array of professionals working with pregnant and parenting adolescents, which likely influenced their perspectives and responses. Similarly, adolescent participants in Mexico were recruited primarily from clinics and hospitals, while those in California were often recruited from community-based organizations and had more varied clinical care. Recruitment for eligible youth participants was more difficult in California due to the additional requirement that the person or a parent must have migrated from Mexico. Furthermore, some eligible participants in California may have elected not to participate due to concerns about disclosing their immigration status, although that was not part of the study. Finally, the involvement of a binational team of researchers with different backgrounds and professional roles may have affected the conduct of the different interviews and focus groups as well as the comfort and responses of the participants. Despite these limitations, these findings can help inform clinical interventions and policies to promote youth-centered and culturally sensitive care and improve the quality of care for this underserved patient population.

More research is needed to ascertain if the key themes found in this study are applicable to the maternity care experiences and perspectives of adolescents in other settings and cultures. Future research should assess if improvements in youth-centered maternity care are associated with improved health outcomes and reductions in C-section rates among adolescents and if experiences vary by type of provider.

## Conclusion

Youth-centered maternity care, in which adolescent patients are fully engaged in decision-making and treated respectfully, can positively impact the experiences of the adolescent as well as shape future healthcare seeking behaviors. Many providers and healthcare professionals often lack the capacity, resources, and infrastructure to offer this type of care. Additional training, enhanced clinical guidelines and accountability, increased diversity in the workforce, and improved healthcare infrastructure may help to address individual and structural challenges. Greater emphasis is needed to address the specific concerns and issues facing pregnant adolescents, particularly in limited-resource settings and immigrant communities.

## Supplementary Information

**Additional file 1.** COREQ_Checklist. COREQ (Consolidated criteria for Reporting Qualitative research) Checklist. Description: Qualitative research checklist.

**Additional file 2.** Youth Questionnaire. Focus Group/Interview Survey Questions. Description: Short socio-demographic survey developed by researchers and not under license was administered to youth participants prior to focus groups and interviews.

**Additional file 3.** Focus Group Tool. PIMSA Focus Group Tool. Description: Focus group guide that was developed by researchers for focus group with youth in California and Mexico.

**Additional file 4.** Interview Guide Adolescents. PIMSA Interview Guide for Adolescents. Description: Interview guide that was developed by researchers for interviews with youth in California and Mexico.

**Additional file 5.** Interview Guide for Healthcare Providers. In-depth interview guide with health providers and other experts in Fresno and Guanajuato. Description: Interview guide that was developed by researchers for interviews with health providers in California and Mexico.

## Data Availability

Full qualitative transcripts are available upon request from the corresponding author.

## Abbreviations

CPS: Child Protective Services
C-section: Cesarean Section
PCMC: Person-Centered Maternity Care
