# Supplementary material for: Youth-centered maternity care: a binational qualitative comparison of the experiences and perspectives of Latina adolescents and healthcare providers
Source: BMC Pregnancy Childbirth. 2021 May 2;21:349. doi: 10.1186/s12884-021-03831-4 (PMC8091497; doi:10.1186/s12884-021-03831-4)
Supplement: Supplementary file 2 — Additional file 2. Youth Questionnaire. Focus Group/Interview Survey Questions. Description: Short socio-demographic survey developed by researchers and not under license was administered to youth participants prior to focus groups and interviews. [file 12884_2021_3831_MOESM2_ESM.docx]

**Focus Group/Interview Survey Questions**

1. **How old are you?**
   - 15
   - 16
   - 17
   - 18
   - 19
   - 20
   - Other: _______
2. **Are you currently in school?**
   - No
   - Yes
3. **What is the highest level of school you have completed or are currently in?**
   - Elementary school
   - Middle school
   - Some high school
   - Finished high school
   - Some college
4. **As a result of this pregnancy/birth, did you leave or postpone school?**
   - No
   - Yes
5. **What is your relationship status?**
   - Single (no boyfriend)
   - I have a boyfriend but we don’t live together
   - Living with boyfriend
   - Married
   - Separated/divorced
   - Other: __________
6. **Before this pregnancy, had you ever been pregnant?**
   - No
   - Yes
   - I’m not sure
7. **How many total children do you have?**
   - 0
   - 1
   - 2
   - 3
   - Other: _______
8. **During this pregnancy, were you diagnosed with any of the following:**

**(Mark all that apply)**

- - Diabetes
  - Gestational diabetes
  - Preeclampsia
  - None of the above
  - I’m not sure
  - Other medical condition:________________________

1. **During this pregnancy, were you ever told by a medical provider (doctor, nurse, nutritionist, etc.) that you were overweight or obese?**
   - No
   - Yes
   - I’m not sure
2. **Have you heard of a clinic or doctor in your community where teens can get sexual health services (such as condoms, birth control pills, pregnancy tests, and STD/HIV tests or information)?**
   - No
   - Yes
   - I’m not sure
3. **Which of the following methods of contraception (birth control) have ever you used?**

**(Mark all that apply)**

- - None
  - Condoms
  - Implant (such as Implanon or Nexplanon)
  - IUD (such as Mirena, ParaGard, Liletta, Kyleena or Skyla)
  - Injectable (such as Depo-Provera)
  - The pill
  - Vaginal ring
  - The patch (such as Xulane)
  - Pulled out before sperm came out (withdrawal)
  - We had sex during the safe time of month (Rhythm method)
  - Emergency contraception (sometimes called EC or morning after pill)
  - Other_________

1. **What was the LAST method of contraception (birth control) you were using prior to this pregnancy?**
   - None
   - Condoms
   - Implant (such as Implanon or Nexplanon)
   - IUD (such as Mirena, ParaGard, Liletta, Kyleena or Skyla)
   - Injectable (such as Depo-Provera)
   - The pill
   - Vaginal ring
   - The patch (such as Xulane)
   - Pulled out before sperm came out (withdrawal)
   - We had sex during the safe time of month (Rhythm method)
   - Emergency contraception (sometimes called EC or morning after pill)
   - Other_________
2. **At what age did you migrate from Mexico? _____**
3. **Since moving to the United States, have you returned to Mexico to live or visit?**
   - **No**
   - **Yes**
4. **Which of the following people that you know have migrated to the United States?**

**(Mark all that apply)**

- - Father of this baby
  - Previous boyfriend
  - Mother
  - Father
  - Sibling (brother, sister)
  - Aunt or uncle
  - Other**: ____________**
  - No one I know has migrated to the United States

1. **Where do the following people currently live?**

|  | **Mexico** | **United States** | **Don’t know** |  |
| --- | --- | --- | --- | --- |
| **Father of this baby** |  |  |  |  |
| **Mother** |  |  |  |  |
| **Father** |  |  |  |  |
| **Sibling (brother, sister)** |  |  |  |  |
| **Aunt or uncle** |  |  |  |  |
| **Other: ___________** |  |  |  |  |

**If you have already had a baby…**

1. **What type of contraception (birth control) do you currently use?**
   - None
   - Condoms
   - Implant (such as Implanon or Nexplanon)
   - IUD (such as Mirena, ParaGard, Liletta, Kyleena or Skyla)
   - Injectable (such as Depo-Provera)
   - The pill
   - Vaginal ring
   - The patch (such as Xulane)
   - Pulled out before sperm came out (withdrawal)
   - We had sex during the safe time of month (Rhythm method)
   - Emergency contraception (sometimes called EC or morning after pill)
   - Other_________
2. **What was the delivery method for this last pregnancy?**
   - Vaginal delivery
   - C-section/caesarean section
3. **Was your baby born pre-term (37 weeks or less)?**
   - No
   - Yes
   - Not sure
