# Supplementary material for: Youth-centered maternity care: a binational qualitative comparison of the experiences and perspectives of Latina adolescents and healthcare providers
Source: BMC Pregnancy Childbirth. 2021 May 2;21:349. doi: 10.1186/s12884-021-03831-4 (PMC8091497; doi:10.1186/s12884-021-03831-4)
Supplement: Supplementary file 3 — Additional file 3. Focus Group Tool. PIMSA Focus Group Tool. Description: Focus group guide that was developed by researchers for focus group with youth in California and Mexico. [file 12884_2021_3831_MOESM3_ESM.docx]

**PIMSA Focus Group Tool**

**Location: Date:**

**Moderator: Notetaker:**

**Number of attendees:**

**INTRODUCTION**

Hello and welcome, we want to thank you for being here. We really appreciate your willingness to share your thoughts with us.

My name is ______________ and I will be leading this discussion. This is _________________ and s/he will be taking notes today. We are researchers at the University of California in San Francisco and the National Perinatology Institute of Mexico. This study is about adolescent pregnancy and the prenatal/postnatal care you received. We are going to ask you some questions about your prenatal/postnatal care, your community, and migration.

The focus group will take about 60 minutes. Before we get started we are going to pass out and read an information sheet that describes what we will be doing today.

*[Give copy of Participant Consent to youth and read aloud. If participants choose not to take part, thank them for coming and make arrangements to provide them with their incentives.]*

**[CONDUCT SURVEY]**

In a group discussion like this it is very important that you express yourself openly – there are no right or wrong answers. We really want to know what **you** think. If you find that you have a different opinion or different experience than the rest of the group, we really would like to hear it, since you represent other young people.

GROUP AGREEMENTS

Before we get started here today I would like to go over some agreements. First, let’s all take a moment to turn our cell phones off:

1. Please speak one at a time and try not to have side conversations as they can be distracting to others and might make others feel uncomfortable. Also, since we are tape recording our conversation, please speak clearly and loud enough for the recorder to capture your voice.
2. We want to hear everyone’s thoughts. If you know you tend to be talkative, keep in mind that others may not be. If you tend to be on the quieter side, please try to speak up because we want to know what you think.
3. Some of the thoughts shared here today may be private. Nothing we say in here today should be shared with anyone outside of this room. While you may not think that some of the things said in here are private, other people might, so please don’t share anything.
4. As we previously mentioned, what you say in this group will be kept confidential and your name will not be linked with what you share.
5. If there is anything you feel uncomfortable sharing with the group, you don’t have to answer.
6. Please listen to other people’s opinions respectfully. We’re all here to share our thoughts, so please, no put-downs or criticism.

Does everyone understand and agree? Does anyone have any other group agreements to add?

Before we get started, does anyone have any questions? [*Answer questions*]

### First, I want to go around and ask everyone to share their name.

### [TURN ON TAPE RECORDERS AND MICROPHONES, TURN MICROPHONE VOLUME ALL THE WAY UP]

### *Community Life*

I want to begin the focus group by asking about the community in which you live.

1. **(*) How would you describe your neighborhood/community?**

[*PROMPT: How would you describe what your neighborhood looks like?]*

1. **(*) How common is it for people in your community to have migrated from Mexico to the United States?**

***[****PROMPT: Do you have family and/or friends who migrated to the United States? Where to? Is it mainly older or younger people who are migrating? More males, females, or both?]*

1. **What are some of the reasons people in your community migrated to the United States? What are some of the reasons for moving to Fresno?**

*[PROMPT: Financial? Familial? Do they migrate for an indefinite period of time or do most people go back to Mexico eventually?]*

1. **Can you tell me about a common migration pattern/story among people in your community? *[****PROMPT: For example, Do people move directly to your community from Mexico, move around the US/CA first? Move seasonally?*
2. **(*) What effect do you think this migration has had on families and communities, whether positive or negative?**

***[****PROMPT: Increased remittances and prosperity to home community/family?]*

1. **What effect does migration have on romantic relationships/couples? Do couples stay together when one of them migrates or relocates to another country/state/city?**
2. **What opportunities are available to you and other youth in your community?**

*[PROMPT: How much schooling do most youth complete? Are there job opportunities? Do youth in your community feel hopeful about the future?]*

1. **Do youth know where they can go to obtain sexual health services and information in the community?**

*[PROMPT: What would keep youth from accessing these services? Cost, transportation, confidentiality, etc.]*

1. **Are there concerns about violence in your community?**

*[PROMPT: Do you feel safe walking around your neighborhood? Domestic violence? Gang violence? What impact do you think this is having on individuals, families, and the community? Are there any services in your communities that you are aware of to address these issues?]*

1. **How common is drug and alcohol use in your community?**

*[PROMPT: Is this common among youth (males and females)? If yes, what do you think are some of the contributing factors that are encouraging youth to engage with drugs? What impact do you think this is having on individuals, families, and the community? Are there any services in your communities that you are aware of to address these issues?]*

### *Health Services*

### Now I would like to ask you some questions about your prenatal and postnatal care experiences.

1. **How do you feel about the available programs and/or activities in health centers or in the community that provide support to pregnant/parenting adolescents?**

**[***PROMPT: Do you think that there are enough programs/activities? Are these activities only for adolescents or for different age groups?*

1. **(*) How would you describe your prenatal care experience?**

[*PROMPT: Did your provider answer all your questions related to your condition? Did you feel comfortable asking them? How many prenatal care visits did you have?]*

1. **(*) What types of services did you receive during your pregnancy?**

[*PROMPT: Did you receive any services on nutrition, weight gain, delivery method, physical activity? Were these provided in the community, health care center, both? How did you hear about them? Were these individual or group activities? Were other adolescents part of these activities?]*

1. **How would you describe your experience during delivery?**

[*PROMPT: Did you have a pre-term birth? Did you have a vaginal delivery or C-section? Whose decision was it (provider, youth, family member, partner, etc.)? Why was this decision made?*]

1. **(*) What type of services have you received after your pregnancy?**

**[***PROMPT:* *Did you attend any postnatal group activities? Did a nurse or community health worker come to your house?]*

1. **(*) Based on your delivery experience and prenatal/postnatal services received, what suggestions or advice do you have for other pregnant/parenting youth? What suggestions or recommendations do you have for health care providers?**

[*PROMPT:* *What would you change? What do you wish you knew or someone had told you in advance?*]

### *Friends and Family*

Now I would like to ask you about your relationship with friends and family.

1. **Among your friends and family, how common is it to get pregnant or to get someone pregnant as a teenager?**
2. **As a result of this pregnancy, what aspects of your life changed?**

***[****PROMPT: Impact on relationship with baby’s father? Schooling/employment? Life goals?]*

1. **How did your family respond to your pregnancy? How about your friends? What about other youth and adults in your community?**

***[****PROMPT: Supportive? Happy? Unhappy? Stigma?]*

Thank you so much for all of your helpful answers. I want to ask you a couple more questions just to make sure I’ve covered everything that you think is important.

1. **(*) Based on your reproductive experience, what do you think young people need from their partner, family, community health services, school, and friends to prevent an unplanned pregnancy?**

*[PROMPT: What advice would you give to other youth to prevent pregnancy, aside from using contraceptives?]*

1. **Now thinking about everything we talked about today, is there anything that we should have covered, but didn’t?**

Thank you for taking the time to participate in our focus group.

**(*) Key questions to ask**

**[FOCUS GROUP DEBRIEF - UCSF STAFF]**

**Process:**

Provide a 1-2 sentence summary of the group.

1. What went well?
2. What could have gone better?
3. Were there any challenges with the group dynamic?
4. How did the conversation flow?
5. Were there any problems with tape recorders, microphones, computers?
6. What should be done differently next time?
7. How long was the group?
8. Was this too much/too little time?
9. Was the time allotted to each question sufficient?

**Content**

1. How were the questions received?
2. Which questions were challenging to deliver?
3. Which questions were challenging for the youth to understand or respond to?
4. Do the responses to the questions provide relevant information?
5. What new, interesting, or surprising information came up?
6. Did anything come up in the focus group that should be added to the tool?
