# Supplementary material for: Youth-centered maternity care: a binational qualitative comparison of the experiences and perspectives of Latina adolescents and healthcare providers
Source: BMC Pregnancy Childbirth. 2021 May 2;21:349. doi: 10.1186/s12884-021-03831-4 (PMC8091497; doi:10.1186/s12884-021-03831-4)
Supplement: Supplementary file 4 — Additional file 4. Interview Guide Adolescents. PIMSA Interview Guide for Adolescents. Description: Interview guide that was developed by researchers for interviews with youth in California and Mexico. [file 12884_2021_3831_MOESM4_ESM.docx]

**PIMSA Interview Guide for Adolescents**

**Location: Date:**

**Interviewer:**

**INTRODUCTION**

Hello and welcome, we want to thank you for being here. We really appreciate your willingness to share your thoughts with us.

My name is ______________ and I will interview you. This interview is part of a study conducted by the University of California in San Francisco and the National Perinatology Institute of Mexico. This study is about adolescent pregnancy and the prenatal/postnatal care you received. We are going to ask you some questions about your prenatal/postnatal care, your community, and migration.

The interview will take around 60 minutes. Here is a research consent form that is yours to keep. If you have any questions or concerns, our contact information is listed on the consent form. We would like to remind you that participation is voluntary, your responses will be confidential, and you don’t have to answer any questions that you don’t want to answer. There is no direct benefit to you but you will receive a gift **($20 gift card in Fresno/a gift in Mexico)** for your participation.

I will take notes during the interview. The interview will be recorded so that I can listen back and improve on my notes. The recording will only be used to be sure we caught everything you said. It will not be shared, and we will destroy the recording once our notes are complete and we have analyzed the data.

*[Give copy of Participant Consent to youth and read aloud. If participant choose not to take part, thank them for coming and make arrangements to provide them with their incentives.]*

Do you have any questions? *[Answer any questions they may have]* Ok, let’s get started.

**Community life**

I would like to start by asking you about your community…

1. How would you describe your neighborhood/community?
2. How common is it for people in your community to have migrated from Mexico to the United States? *[PROMPT: Do you have family and/or friends who migrated to the United States? Where to? Is it mainly older or younger people who are migrating? More males, females, or both?]*
3. What are some of the reasons people in your community migrated to the United States? What are some of the reasons for moving to Fresno? [*PROMPT: Financial? Familial? Do they migrate for an indefinite period of time or do most people go back to Mexico eventually?]*
4. What opportunities are available to you and other youth in your community?

*[PROMPT: How much schooling do most youth complete? Are there job opportunities? Do youth in your community feel hopeful about the future?]*

1. Do youth know where they can go to obtain sexual health services and information in the community? [*PROMPT: What would keep youth from accessing these services? Cost, transportation, confidentiality, etc*.]
2. How common is it in your community for teenagers to get pregnant?

**Experience with health care services during the pregnancy**

Now I would like to ask you about your prenatal care…

1. How would you describe your prenatal care experience?
2. Did you experience any problem or illness during your pregnancy? *[If she does not mention spontaneously ask for eclampsia, hypertension, diabetes, obesity, etc]?*
3. What type of services did you receive during your pregnancy to avoid complications related with _________ *[any co-morbidity that she mentioned]*
4. How was the support by the hospital staff in managing your disease? How was the interaction with the staff?
5. Did you receive any support from services in the community during your pregnancy? Which ones? What was your opinion of those services? Would you recommend them to other teens?
6. What suggestions or recommendations do you have for improving services for pregnant and parenting adolescents?
7. What type of information did the clinic staff give you about your delivery? Did they mention different types of delivery (C-section, vaginal)?
8. Did you have a C-section or vaginal delivery? Was that your choice?
9. Did you have a pre-term birth (less than 37 weeks)? How did you feel about it?

**Experience with health care services after the pregnancy**

Now I would like to ask you about care you received after your baby was born…

1. Since having your baby, what type of postnatal care have you received? [*If she had a complication, ask about follow up, additional nutritional support, etc*.]
2. Are you involved in any parenting group or receiving support at school or with another organization (such as Teen Success)?

**Opinion and experiences with contraceptive use before the pregnancy**

Now I would like to ask you about your contraception use before pregnancy…

1. Prior to your pregnancy, were you using any type of contraception (birth control)? What method were you or your partner using? How did you decide to use that method? Where did you get it? Who gave you information or counseling?

*[If she does not provide information spontaneously, ask about modern and traditional methods and dual method use (birth control and condoms. If she tried several methods register the information]*

1. If you were not using a method, why was that? Did you make the decision not to use a contraceptive by yourself? *[If she does not mention spontaneously, asked if her decision was related with her health conditions: obesity, diabetes, hypertension]*
2. What was your partner’s involvement and opinion about using contraception before and after the last pregnancy?

**Opinion and experiences with contraceptive use after the pregnancy**

Now I would like to ask you about your contraception use after pregnancy…

1. Did you decide to use contraception after the delivery (C-section or vaginal delivery)? What type did you choose and why? Did anyone provide information or counseling about this or other contraceptive methods? Are you still using the same method? *[Explore different reasons for not using contraception: side effects, obesity, diabetes, cost, partner, family, breastfeeding]*
2. What is your opinion about getting pregnant in the future?

**Migration and family relationships**

Now I would like to ask you about your personal experience with migration as well as your relationship with your family…

1. Why did you and/or your family first move to the United States? Did you and your family move directly to Fresno County or have you lived in other parts of California or the United States? *[Please describe your migration pattern/route.]*
2. What impact has your migration to the US had on you and your family? [*if she doesn’t mention spontaneously, explore positive and negative consequences*]
3. How did your family respond to your pregnancy? How about your friends? What about other youth and adults in your community? [*Supportive? Happy? Unhappy? Disappointed?*]?
4. What type of support did you receive from your family during your pregnancy? Now that you had your baby?
5. In your family, did either of your parents or any of your brothers or sisters have children while they were teenagers?
6. Prior to this pregnancy, what was your relationship with your baby’s father [*long-term couple, first partner, etc.*]? Did you ever talk about using contraception [*explore why or why not*]? Did you ever talk about having kids?
7. Since having your baby, what is your relationship with your baby’s father? Are you still romantically involved? Is he still involved with parenting or providing other support?
8. Did he migrate to the US? If yes, what was the reason he migrated? Where did he migrate to in the US?
9. Who do you currently live with? Do you live with your partner, his family, or your family?
10. Finally, based on your experiences during this pregnancy and after delivery, what do young people need from people around them to achieve healthy reproductive health outcomes such as preventing an unplanned pregnancy?

**Thank you for taking the time to participate in the interview.**
