# Supplementary material for: Youth-centered maternity care: a binational qualitative comparison of the experiences and perspectives of Latina adolescents and healthcare providers
Source: BMC Pregnancy Childbirth. 2021 May 2;21:349. doi: 10.1186/s12884-021-03831-4 (PMC8091497; doi:10.1186/s12884-021-03831-4)
Supplement: Supplementary file 5 — Additional file 5. Interview Guide for Healthcare Providers. In-depth interview guide with health providers and other experts in Fresno and Guanajuato. Description: Interview guide that was developed by researchers for interviews with health providers in California and Mexico. [file 12884_2021_3831_MOESM5_ESM.docx]

**In-depth interview guide with health providers and other experts**

**in Fresno and Guanajuato**

**Location: Date:**

**Interviewer: Notetaker:**

**Interviewee: Organization:**

Hello and welcome, I want to thank you for participating in this interview. I really appreciate your willingness to share your thoughts with me.

My name is ______________. This interview is part of a study conducted by the University of California in San Francisco and the National Perinatology Institute of Mexico. This study is about adolescent pregnancy and the prenatal/postnatal care they received. We also want to explore the relationships between pregnancy and migration between Mexico and the US. So, please think about the population of pregnant adolescents with a history of migration when you answer these questions.

Some of these questions will relate directly to your work, but there may be topics that you don’t know about or don’t feel comfortable answering. If that’s the case, please just tell me, and we’ll move on. We will be interviewing other experts in the community, so please don’t feel that you need to give us an answer if you’re uncertain about a particular topic.

The interview will last about 60 minutes. Here is a research consent form that is yours to keep. If you have any questions or concerns, our contact information is listed on the consent form. We would like to remind you that participation is voluntary, your responses will be confidential, and you don’t have to answer any questions that you don’t want to answer. You will receive a $20 gift card for your participation.

I will take notes during the interview. The interview will be recorded so that I can listen back and improve on my notes. The recording will only be used to be sure we caught everything you said. It will not be shared, and we will destroy the recording once our notes are complete and we have analyzed the data.

Do you have any questions? *[Answer any questions they may have]* Ok, let’s get started.

1. In your opinion, what impact does migration between Mexico and the US have on adolescent pregnancy? What about other health complications that you see among adolescents such as obesity and diabetes?
2. Based on what you have observed, do young women generally move to the United States with family? By themselves? With a partner?
3. What factors at the community, family, school, and interpersonal level do you think are influencing the occurrence of pregnancy? What about for obesity and diabetes? ***[For interviewees in Mexico ask for factors affecting adolescents who themselves or their partners have a history of migration? For interviewees in Fresno ask specifically for factors affecting Mexican migrants]***
4. For the population of adolescents you serve, do they continue to move [around the state or country or between the US and Mexico]? How does this affect their continuation of care?
5. For the pregnant and parenting adolescents you work with, how does migration impact their relationship with their partners? [**For example, is the partner generally Latino, do they stay together, does one move for work**?]
6. Do you conduct specific activities for pregnant adolescents (e.g. centering pregnancy model/group prenatal care)? How are they different from prenatal care services for adult women?
7. Could you tell me about the services that you provide to pregnant adolescents to prevent adverse pregnancy outcomes such as gestational diabetes, preeclampsia, pre-term birth, or C-sections? What about programs or services targeting adolescents with substance use or victims of violence? ***[For interviewees in Fresno ask, specifically about services for Mexican migrants]***
8. Are there any protocols of attention **[for Mexico: Normas mexicanas de atencion del embarazo, parto y puerperio de la SSA y otras guias hospitalarias internas]** that you follow in the clinic for pregnant adolescents? Do you face any barriers to implement them? ***[Only for clinicians]***
9. Could you describe the support programs for adolescents in pre-natal care that you have in the clinic/organization? How often do they meet, how many hours per week, how many participants do they have? Do you follow a cohort of women? ***[For interviewees in Fresno ask, specifically about services for Mexican migrants]***
10. Do you know about other programs, activities, resources in the community that support pregnant adolescents? Does your clinic/ organization work in coordination with them? ***[For interviewees in Fresno ask specifically about resources, activities for Mexican migrants]***
11. What types of services are available for postnatal or parenting adolescents?
12. Could you describe any barriers that adolescents face to access contraception in the community? In your opinion, are there specific barriers that these adolescents face? ***[For interviewees in Fresno ask, specifically about barriers that Mexican migrants face] [For interviewees in Guanajuato, ask about barriers that adolescents whose partners migrate to the US face]***
13. Do you have any recommendations to improve prenatal and postnatal services for adolescents in your community who have a history of migration? ***[For interviewees in Mexico ask for recommendations for adolescents who themselves or their partners have a history of migration? For interviewees in Fresno ask specifically for recommendations affecting Mexican migrants]***
14. Is there anything else that we didn’t cover that you would like to add?

Thank you so much for your time.
